# Establishment and Validation of a Ferroptosis-Related Long Non-Coding RNA Signature for Predicting the Prognosis of Stomach Adenocarcinoma

**DOI:** 10.3389/fgene.2022.818306

**Published:** 2022-02-15

**Authors:** Shuqiong Zhang, Naisheng Zheng, Xiaocui Chen, Kun Du, Junyao Yang, Lisong Shen

**Affiliations:** ^1^ Department of Clinical Laboratory, Xinhua Hospital, Shanghai Jiao Tong University School of Medicine, Shanghai, China; ^2^ Faculty of Medical Laboratory Sciences, Shanghai Jiao Tong University School of Medicine, Shanghai, China; ^3^ Xin Hua Children’s Hospital, Shanghai, China

**Keywords:** ferroptosis, long non-coding RNA, stomach adenocarcinoma, LINC01615, nomogram

## Abstract

**Background:** Ferroptosis is a form of regulated cell death that follows cell membrane damage and mostly depends on iron-mediated oxidative. Long non-coding RNAs (LncRNAs) are associated with the development of a variety of tumors. Till date, LncRNAs have been reported to intervene in ferroptosis. Therefore, we intended to provide a prognostic ferroptosis-related-lncRNA signature in stomach adenocarcinoma (STAD).

**Methods:** We downloaded ferroptosis-related genes from the FerrDb database and RNA sequencing data and clinicopathological characteristics from The Cancer Genome Atlas. Gene differential expression analysis was performed using the “limma” package. We used Cox regression analysis to determine the ferroptosis-related lncRNAs signature with the lowest AIC value. The Kaplan–Meier curve, ROC curve, and nomogram were used to evaluate the prognostic value of the risk score. Gene set enrichment analysis (GSEA) was used to explore the biologic functions of the three ferroptosis-related lncRNAs. LINC01615 expression in gastric cancer cell lines and tissues was measured by real-time PCR. A nuclear-cytoplasmic fractionation assay was used to analyze the subcellular localization for LINC01615. Furthermore, we used bioinformatics to predict potential target microRNAs (miRNAs) of LINC01615 and their target ferroptosis-related mRNAs.

**Results:** Three ferroptosis-related-lncRNA signatures (AP000695.2, AL365181.3, and LINC01615) were identified, and then Kaplan–Meier, Cox regression analyses, and ROC curve confirmed that the ferroptosis-related-lncRNA model could predict the prognosis of STAD. The GSEA indicated that the three ferroptosis-related lncRNAs might be related to the extracellular matrix and cellular activities. LINC01615 is highly expressed in gastric cancer cell lines and tissues. A nuclear-cytoplasmic fractionation assay confirmed that in gastric cancer cell lines, most LINC01615 was enriched in the cytoplasm. Bioinformatics further predicts four potential target miRNAs of LINC01615 and then figured out 26 target ferroptosis-related mRNAs.

**Conclusion:** We established a three-ferroptosis-related-lncRNA model (AP000695.2, AL365181.3, and LINC01615) that can predict the prognosis of STAD patients. We also expected to provide a promising target for LINC01615 for research in the future, which was highly expressed in gastric cancer and cell lines and acted as a ceRNA to get involved in ferroptosis.

## Introduction

Stomach cancer is still a worldwide issue in 2020, ranking fifth for incidence and fourth for mortality globally (Sung et al., 2021). Although screening and treatment methods for stomach cancer have improved, the survival rate of advanced gastric cancer is still low. Personalized cancer treatment has become an emerging research hotspot with the advancement of medicine. Therefore, more comprehensive and accurate markers for gastric cancer diagnosis and monitoring are yet to be discovered.

Long non-coding RNAs (lncRNAs) are transcripts longer than 200 nucleotides that have no or limited protein-coding capacity (Gutschner and Diederichs, 2012). LncRNAs can interact with proteins, RNAs, and DNAs to get involved in almost all biological processes and development of various diseases such as chromatin kinetics, RNA processing, protein synthesis, cell growth, apoptosis, and ferroptosis (De Los Santos et al., 2019). Ferroptosis, as a unique, nonapoptotic modality of cell death, is primarily caused by intracellular iron catalytic activity and lipid peroxidation (Chen et al., 2021). It is modulated by lipid repair systems such as glutathione and GPX4 and relies on the biosynthesis of PUFA-containing phospholipids (Stockwell et al., 2017). Ferroptosis may be a potential immune surveillance of cancer, although the exact mechanism leading to the ferroptotic cell death is still unclear, and further studies are required to validate the functions (Jiang et al., 2021).

Till date, there have been many studies about the relationship between lncRNA and ferroptosis. For example, the p53-related lncRNA P53RRA can directly interact with the signal protein to activate the p53 pathway (Mao et al., 2018). LncRNA ZFAS1(Yang et al., 2020), PVT1 (Lu et al., 2020), MT1DP (Gai et al., 2020), OIP5-AS1 (Zhang et al., 2021), and LINC00336(Wang et al., 2019) can interact with miRNA as competitive endogenous RNA (ceRNA), thereby affecting the expression of ferroptosis-related mRNAs. Accordingly, it is necessary to identify ferroptosis-related lncRNAs to predict the prognosis of STAD patients and offer a basis for individualized treatment. In this study, we constructed a three-ferroptosis-related-lncRNA (AP000695.2, AL365181.3, and LINC01615) signature-based nomogram to quantify a STAD patient’s probability of overall survival (OS). The study may result in predicting survival and formulating individualized treatments in STAD patients.

## Materials and Methods

### Data Source

The RNA expression profiles and the clinical data of 375 STAD patients and 32 normal control patients from the publicly available TCGA-STAD Project were downloaded from the Genomic Data Commons Data Portal (https://portal.gdc.cancer.gov/, accessed 22 August 2021). We searched The FerrDb database (http://www.zhounan.org/ferrdb; accessed 26 August 2021) to identify 259 genes involved in ferroptosis.

### Bioinformatics Analysis for the Construction of Ferroptosis-Related LncRNA Prognostic Model

Gene differential expression analysis was performed using the “limma” package. We drew volcano plots and used R software with the “ggplot2” package. Heatmaps were drawn using the “pheatmap” package. The “rms” package was utilized to establish a prognostic nomogram combined with a three-ferroptosis-related-lncRNA model and clinical risk factors. The potential biologic functions of three ferroptosis-related lncRNAs were explored with gene set enrichment analysis (GSEA). DIANA, miRDB, and RNA22 v2 databases were used to predict potential target microRNAs, while miRDB, Targetscan, and DIANA-Tarbase were used to figure out the miRNAs target genes. Protein–protein interaction (PPI) networks were drawn with the STRING database.

### Clinical Tissue Samples

Gastric cancer tissues and paracancerous tissues were obtained from 25 patients at Xinhua Hospital of Shanghai Jiao Tong University School of Medicine. All tissues were immediately frozen in liquid nitrogen and stored at −80°C until RNA extraction. The study was approved by the Institutional Review Board and Ethics Committee of Xinhua Hospital of Shanghai Jiao Tong University School of Medicine, and it was performed under the ethical guidelines of the Declaration of Helsinki.

### Quantitative Real-Time PCR

Total RNAs from cells and tissue samples were isolated using TRIzol reagent (Invitrogen, Carlsbad, CA, USA). Then RNA was reversely transcribed to cDNA using a PrimeScript™ RT Reagent Kit (TaKaRa, Shiga, Japan). cDNA was used for real-time PCR assays utilizing qPCR SYBR Green Master Mix (YEASEN, Shanghai, China). Results were normalized to the expression of GAPDH. The PCR primer sequences were: LINC01615 forward: 5′-AAG​ACA​GGG​GAT​CCC​GAA​GA-3′, reverse: 5′-CAG​GAT​TTG​GGC​ATC​TCG​GT-3’; GAPDH forward: 5′-TTG​GTA​TCG​TGG​AAG​GAC​TCA-3′, reverse: 5′-TGT​CAT​CAT​ATT​TGG​CAG​GTT​T-3’; U6 forward: 5′- TGG​AAC​GCT​TCA​CGA​ATT​TGC​G -3′, reverse: 5′- GGA​ACG​ATA​CAG​AGA​AGA​TTA​GC -3’.

### Cell Lines and Culture Condition

Human gastric mucosa epithelial cell (GES-1) and gastric cancer cell lines (MKN-45, HGC-27, and AGS) were obtained from the Chinese Academy of Sciences (Shanghai, China) and cultivated in Dulbecco’s modified Eagle medium with 10% FBS (Gibco, Grand Island, NY, USA) in the condition of 5% CO_2_ at 37°C.

### Subcellular RNA Fractionation

The PARIS™ Kit (Ambion, Austin, TX) was used to isolate RNA from nuclear and cytoplasmic fractions of gastric cancer cell lines. Then, we used GAPDH and U6, respectively, as cytoplasmic and nuclear controls for further analysis with PCR.

### Statistical Analysis

The statistical analyses were done using R software (version 4.1.1) or GraphPad Prism 8.0 (GraphPad, La Jolla, CA, USA). Ferroptosis-related lncRNAs were identified by Pearson correlation analysis. The survival difference was calculated using the Kaplan–Meier method. The specificity and sensitivity of the prognostic prediction were performed using a time-dependent ROC curve. A nomogram was constructed with the lncRNA model and clinical risk factors by multivariate Cox analysis. To validate the nomogram, the C-index and calibration plot were done using R software. For the further assays about LINC01615 *in vitro*, differences between the two groups were analyzed with Student’s *t*-test. A *p*-value <0.05 (two-tailed) was considered statistically significant.

## Results

### Identified Ferroptosis-Related LncRNAs in the TCGA-STAD Project

**FIGURE 1:**
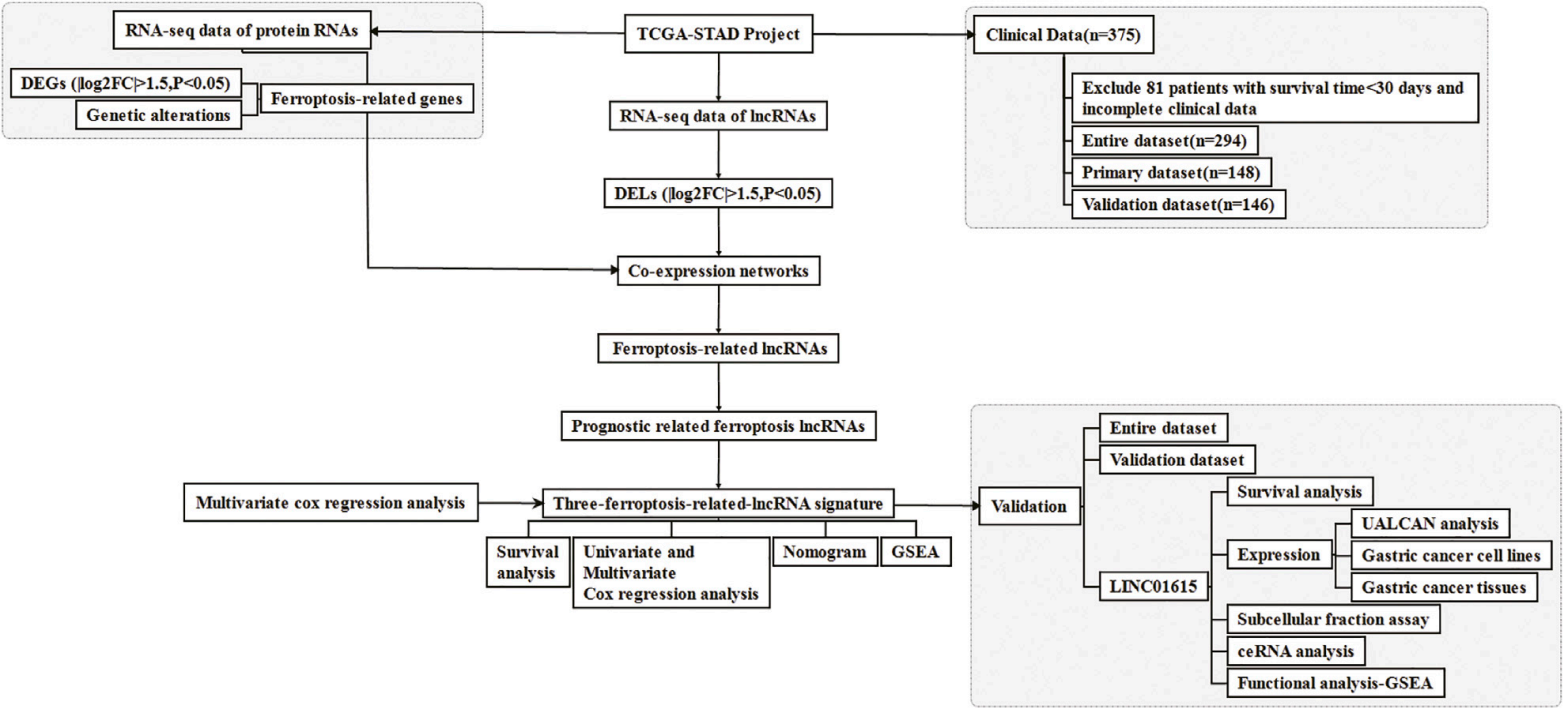
Flow chart for the research.

**FIGURE 2:**
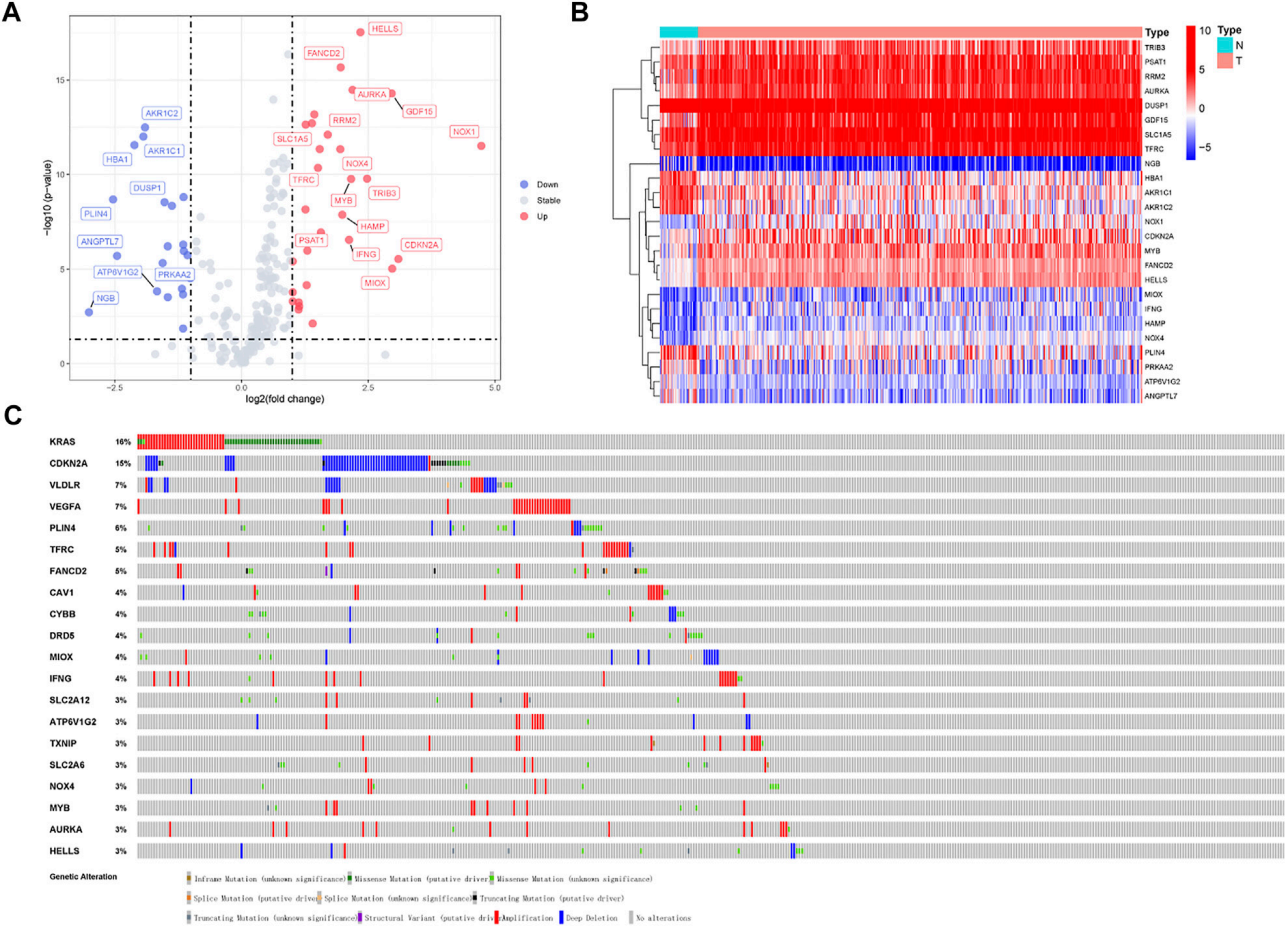
Volcano plot and heatmap of ferroptosis-related genes of stomach adenocarcinoma samples in TCGA-STAD Project. **(A)** Volcano plot of 259 ferroptosis-related genes in TCGA-STAD Project. **(B)** Heatmap of 25 differentially expressed ferroptosis-related genes (log2|fold change| >1.5). **(C)** Mutations in differentially expressed ferroptosis-related genes. 20 genes have a mutation rate ≥ 3%.

A flow chart of the research is shown in Figure 1. We downloaded RNA-seq of 375 tumor tissue samples and 32 nontumor samples from the TCGA-STAD project. The expression of 259 ferroptosis-related genes was fetched from the TCGA-STAD project. We used the “limma” package in R to identify 25 differentially expressed ferroptosis-related genes between STAD samples and normal samples, with a |log2FC| >1.5 and a *p*-value <0.05. Of the 25 differentially expressed genes (DEGs), 16 genes (NOX1, CDKN2A, MIOX, GDF15, TRIB3, HELLS, AURKA, MYB, IFNG, HAMP, FANCD2, NOX4, RRM2, PSAT1, SLC1A5, and TFRC) were found to be upregulated and 9 (DUSP1, PRKAA2, ATP6V1G2, AKR1C2, AKR1C1, HBA1, ANGPTL7, PLIN4, and NGB) were downregulated in the STAD tissues (Supplementary Table S1). The volcano plot and heatmap showed the expression patterns of these DEGs between tumor and nontumor tissues (Figures 2A,B). In addition, the above 25 upregulated and downregulated genes were verified to be consistent with the analysis combined with TCGA and GTEx (Supplementary Table S5).

Mutations of various tumor suppressor genes and oncogenes will activate multiple signal pathways and promote the occurrence and development of tumors. Owing to the great clinical significance of these ferroptosis-related genes, we also explored their genetic alterations (Figure 2C). 20 genes mutate at a rate ≥ 3%, of which KRAS was the most frequently mutated gene (16%), followed by CDKN2A (15%). Mutations in the KRAS gene can activate RAS protein and transforms signals from EGFR to mitogen activated protein kinases (MAPKs), thereby causing cellular proliferation, migration, and drug resistance (Drosten and Barbacid, 2020). CDKN2A is frequently mutated in gastric cancer, resulting in cell invasion, migration, and colony formation. The CDKN2A protein takes part in the cell cycle and senescence through the regulation of the cyclin-dependent kinase (CDK) 4/6 and cyclin D complexes (Zhao et al., 2016).

In addition, we obtained 1943 differentially expressed lncRNAs (DELs) from the TCGA-STAD project, with a |log2FC| >1.5 and a *p*-value <0.05 (Supplementary Table S2). The result was shown with a volcano plot (Supplementary Figure S1A). Then, to make a co-expressed network, we analyzed the correlation between the expression levels of ferroptosis-related genes and differentially expressed lncRNAs in STAD samples with Pearson’s correlation analysis. The heatmap of the 413 ferroptosis-associated lncRNAs is shown in Supplementary Figure S1B. The details of these lncRNAs are offered in Supplementary Table S3.

### Construction and Validation of a Three-Ferroptosis-Related-LncRNA Prognostic Model

We downloaded clinical data from TCGA and excluded 81 patients who lacked some clinical features. Then, the remaining 294 patients were included in the “entire dataset,” and 148 STAD patients were randomly assigned as the “primary dataset,” while the other 146 were the “validation dataset.” There was no statistical difference in the clinical characteristics between the primary and validation datasets. Specific clinical data are shown in Table 1.

**TABLE 1:** Baseline clinical characteristics of STAD patients involved in this study.

Characteristic	Entire dataset	Primary dataset	Validation dataset	*p* value
	*n* = 294	*n* = 148	*n* = 146	
Age (years)				0.965
≥65	166 (56.46%)	86 (58.11%)	80 (54.79%)	
＜65	128 (43.54%)	62 (41.89%)	66 (45.21%)	
**Gender**				**0.490**
Female	185 (62.93%)	96 (64.86%)	89 (60.96%)	
Male	109 (37.07%)	52 (35.14%)	57 (39.04%)	
**TNM stage**				**0.780**
I	37 (12.59%)	18 (12.16%)	19 (13.01%)	
II	97 (32.99%)	50 (33.78%)	47 (32.19%)	
III	130 (44.22%)	67 (45.27%)	63 (43.15%)	
IV	30 (10.20%)	13 (8.78%)	17 (11.64%)	
**Tumor stage**				**0.939**
T1	13 (4.42%)	7 (4.73%)	6 (4.11%)	
T2	60 (20.41%)	29 (19.59%)	31 (21.23%)	
T3	144 (48.98%)	73 (49.32%)	71 (48.63%)	
T4	77 (26.19%)	39 (26.35%)	38 (26.03%)	
**Distant metastasis**				**0.607**
M1	18 (6.12%)	8 (5.41%)	10 (6.85%)	
M0	276 (93.88%)	140 (94.59%)	136 (93.15%)	
**Lymph node metastasis**				**0.890**
N0	89 (30.27%)	45 (30.41%)	44 (30.14%)	
N1	80 (27.21%)	44 (29.73%)	36 (24.66%)	
N2	63 (21.43%)	25 (16.89%)	38 (26.03%)	
N3	62 (21.09%)	34 (22.97%)	28 (19.18%)	

The bold values are the p-value of clinical characteristics between the primary and validation datasets (all p > 0.05). They can be changed to regular formal.

Ferroptosis-associated lncRNAs were analyzed with univariate Cox regression analysis. Thirty-two ferroptosis-related lncRNAs were associated with prognosis (Supplementary Figure S2). We further identified lncRNAs for a model with multivariate Cox regression analysis in R software. The three ferroptosis-related lncRNAs (AP000695.2, AL365181.3, and LINC01615) were identified and integrated into a prognostic model according to their risk coefficients (Table 2). The formula was as follows: Risk Score = (0.454 × Expression AP000695.2) + (−0.081 × Expression AL365181.3) + (0.791 × Expression LINC01615).

**TABLE 2:** Three ferroptosis-related lncRNAs related to OS in the primary dataset.

Gene name	Coefficient	HR	95%CI	*p* value
AP000695.2	0.453658	1.574059	1.05–2.35	0.02713
AL365181.3	−0.0805	0.922653	0.86–0.99	0.021365
LINC01615	0.791086	2.205791	1.23–3.95	0.007804

Next, we used the formula above to get the risk score of every STAD patient in the primary dataset. We divided the primary dataset into a high-risk group (*n* = 74) and a low-risk group (*n* = 74), based on the median risk score. Kaplan–Meier curve analysis indicated that the prognosis of the high-risk group is worse than that of the low-risk group (*p*-value = 5.772E-03) (Supplementary Figure S3A). The distributions of the risk scores and OS statuses in the primary dataset are shown in Supplementary Figures S3B,C. The forest plot of univariate and multivariate Cox regression also illustrated that the risk score is related to OS of STAD patients (Supplementary Figures S3D,E). Subsequently, we use receiver operating characteristic (ROC) curve analysis to quantify the ability of the risk score to predict OS of STAD patients in the primary dataset. The area under the time-dependent ROC curve (AUC) of the three-ferroptosis-related-lncRNA model was 0.675 at 3 years and 0.670 at 5 years. (Supplementary Figures S3F,G).

**FIGURE 3:**
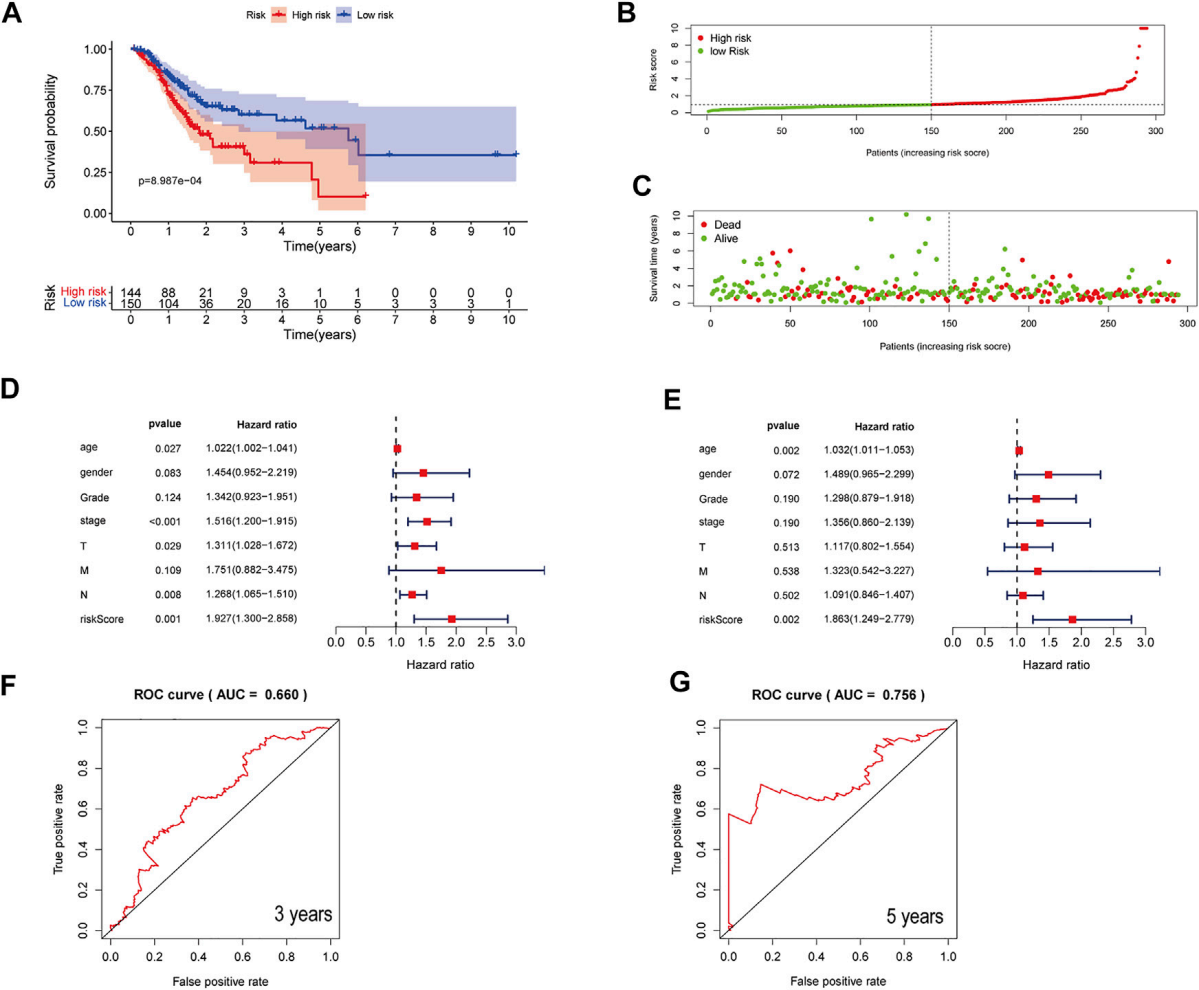
Assessment of a three-ferroptosis-related-lncRNA model for predicting prognosis of STAD in the entire dataset. **(A)** Kaplan–Meier curves of the high-risk and the low-risk groups in the entire dataset. **(B)** The distribution of patients in different risk groups. **(C)** Survival status of patients in the high-risk and the low-risk groups. **(D)** A forest plot of univariate Cox regression analysis in the entire dataset. **(E)** A forest plot of multivariate Cox regression analysis in the entire dataset. **(F, G)** Three- and 5-year ROC curves illustrating the predictive accuracy of the model for prognosis in the entire dataset.

Also, the predictive ability of the three-ferroptosis-related-lncRNA model was verified in the entire dataset (Figure 3) and validation dataset (Supplementary Figure S4). The results in the entire dataset and validation dataset were consistent with the results in the primary dataset. The OS of STAD patients was longer in the low-risk group than in the high-risk group with Kaplan–Meier curve analysis. *p*-value was 8.987e-04 in the entire dataset (Figures 3A–C) and 1.867E-02 in the validation dataset (Supplementary Figure S4A). Forest plot of univariate and multivariate Cox regression also identified that the three-ferroptosis-related-lncRNA model is associated with overall survival (Figures 3D,E, Supplementary Figures S4D,E). The AUC of the three-lncRNA signature was 0.660 at 3 years and 0.756 at 5 years in the entire dataset (Figures 3F,G), while it was 0.656 at 3 years and 0.777 at 5 years in the validation dataset (Supplementary Figures 4F,G).

Therefore, the three-ferroptosis-related-lncRNA model can predict the OS of STAD patients well in both primary and validation datasets.

### A Nomogram Integrating the Clinical Risk Factors With Model

**FIGURE 4:**
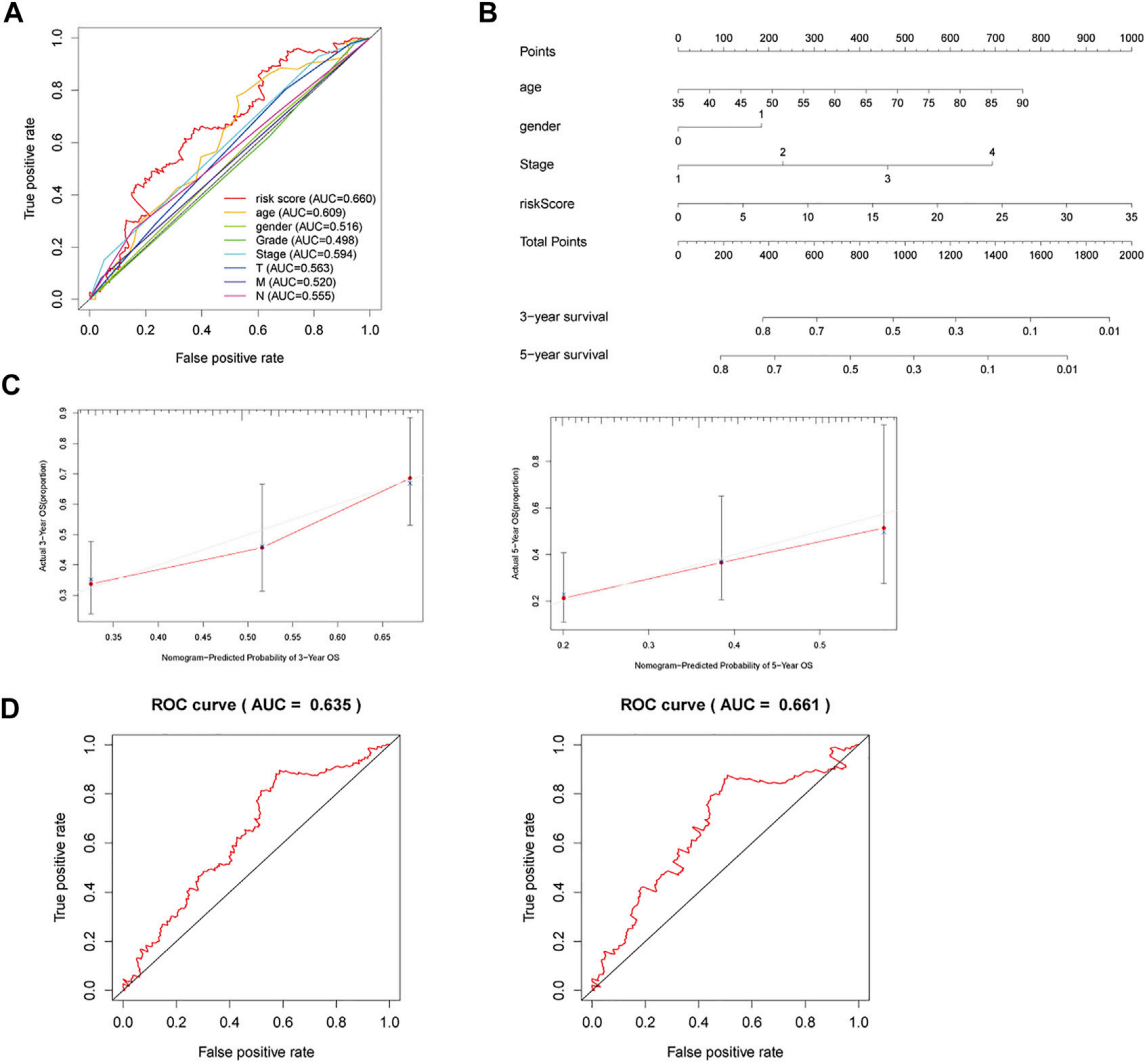
A nomogram to predict three- and 5-year OS for STAD patients. **(A)** ROC curve analysis of risk scores and other clinical risk factors in the entire dataset. **(B)** Nomogram for predicting three- and 5-year OS in STAD patients. **(C)** Calibration plots of the nomogram for predicting three- and 5-year OS in the entire dataset. **(D)** Time-dependent ROC curves of the nomogram for predicting OS in the entire dataset.

Clinical risk factors such as age, the tumor stage, and lymph node metastasis are also important for the prognosis of STAD patients. ROC curve analysis of risk scores and other clinical risk factors in the entire dataset are shown in Figure 4A. The AUC value of the risk score is 0.660 in the entire dataset (Figure 4A), 0.675 in the primary dataset (Supplementary Figure S5A), and 0.767 in the validation dataset (Supplementary Figure S6A). Consequently, we combined the risk score of the three ferroptosis-related lncRNAs with these clinical risk factors to develop a nomogram as an efficient quantitative method to predict individualized prognosis in the primary dataset (Supplementary Figure S5) and validated it in the entire and validation datasets. The nomogram can help us predict the three- and 5-year OS probability of every STAD patient conveniently (Figures 4B,C). We further evaluated the nomogram with a concordance index (C-index), calibration plots, and time-dependent ROC curve analysis. The C-index was 0.635 for the entire dataset, 0.637 for the primary dataset, and 0.666 for the validation dataset. The calibration curve also revealed consistent predicted and actual survival rates in the primary dataset (Supplementary Figure S5B), entire dataset (Figure 3B), and validation dataset (Supplementary Figure S6B).

The three- and 5-year AUC of the nomogram was 0.635 and 0.661, respectively, in the entire dataset (Figure 4D). The three- and 5-year AUC was 0.637 and 0.721, respectively, in the primary dataset (Supplementary Figure S5C), while it was 0.620 and 0.599, respectively, in the validation dataset (Supplementary Figure S6C). Therefore, the nomogram can serve for predicting the prognosis of STAD patients.

### GSEA Between the High- and Low-Risk Groups for Potential Biologic Function

**FIGURE 5:**
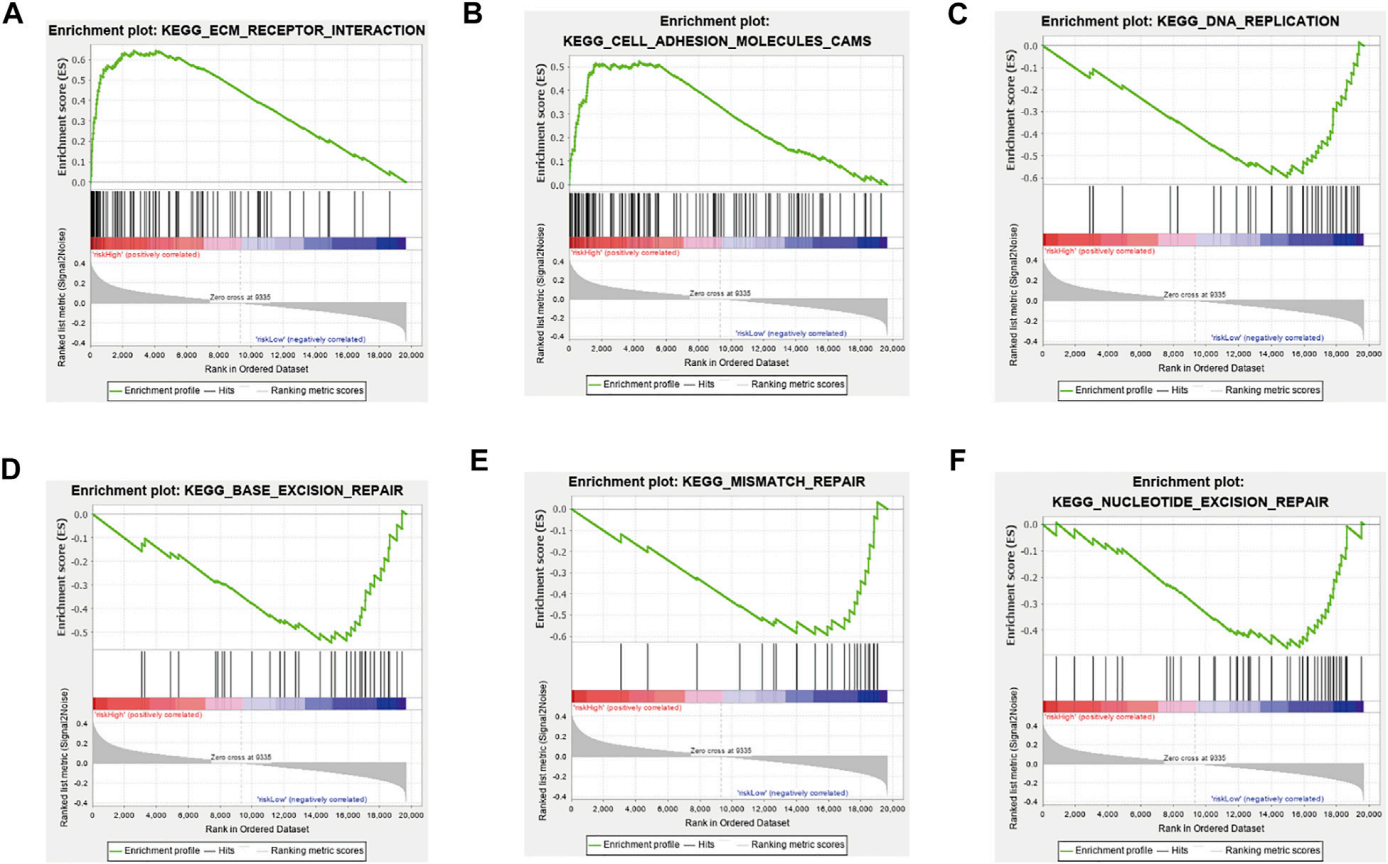
Gene set enrichment analysis between the high-risk group and low-risk group. **(A)** ECM receptor interaction, **(B)** cell adhesion molecules (CAMs) were enriched in the high-risk group, **(C)** DNA replication, **(D)** base excision repair, **(E)** mismatch repair, and **(F)** nucleotide excision repair was enriched in the low-risk group. All |NES|>1, NOM *p*-value < 0.05, FDR q-value < 0.25.

Next, we utilized GSEA to explore the biologic function of the three-ferroptosis-related-lncRNA model. The results of GSEA presented that ECM receptor interaction and cell adhesion molecules (CAMs) were enriched in the high-risk group, while DNA replication, base excision repair, mismatch repair, and nucleotide excision repair were enriched in the low-risk group. Therefore, the three ferroptosis-related lncRNAs may influence the extracellular matrix and cellular activities such as cell adhesion, migration, and DNA replication (Figures 5A–F).

### LINC01615 Expression in Gastric Cancer Cell Lines and Tissues

The fold change of LINC01615 is the highest among three ferroptosis-related lncRNAs. According to the co-expression networks, LINC01615 was positively co-expressed with several ferroptosis-related genes. The detail of co-expression networks between LINC01615 and ferroptosis-related genes are offered in Supplementary Table S6. LINC01615 was positively co-expressed with CAV1, KRAS, and GPX4 (Supplementary Figure S8). CAV1 can promote cancer progression *via* inhibiting ferroptosis in head and neck squamous cell carcinoma (Lu et al., 2022) and drives the execution of acute immune-mediated hepatic damage (Deng et al., 2020). KRAS can promote the generation of ROS, thereby promoting the accumulation of oxidative by-products that decrease the threshold of cancer cells to undergo ferroptosis (Bartolacci et al., 2021). GPX4 can reduce phospholipid hydroperoxide to hydroxy phospholipid, thus inhibiting ferroptosis in cancer cells (Yang et al., 2014). Therefore, we selected LINC01615 for further research.

**FIGURE 6:**
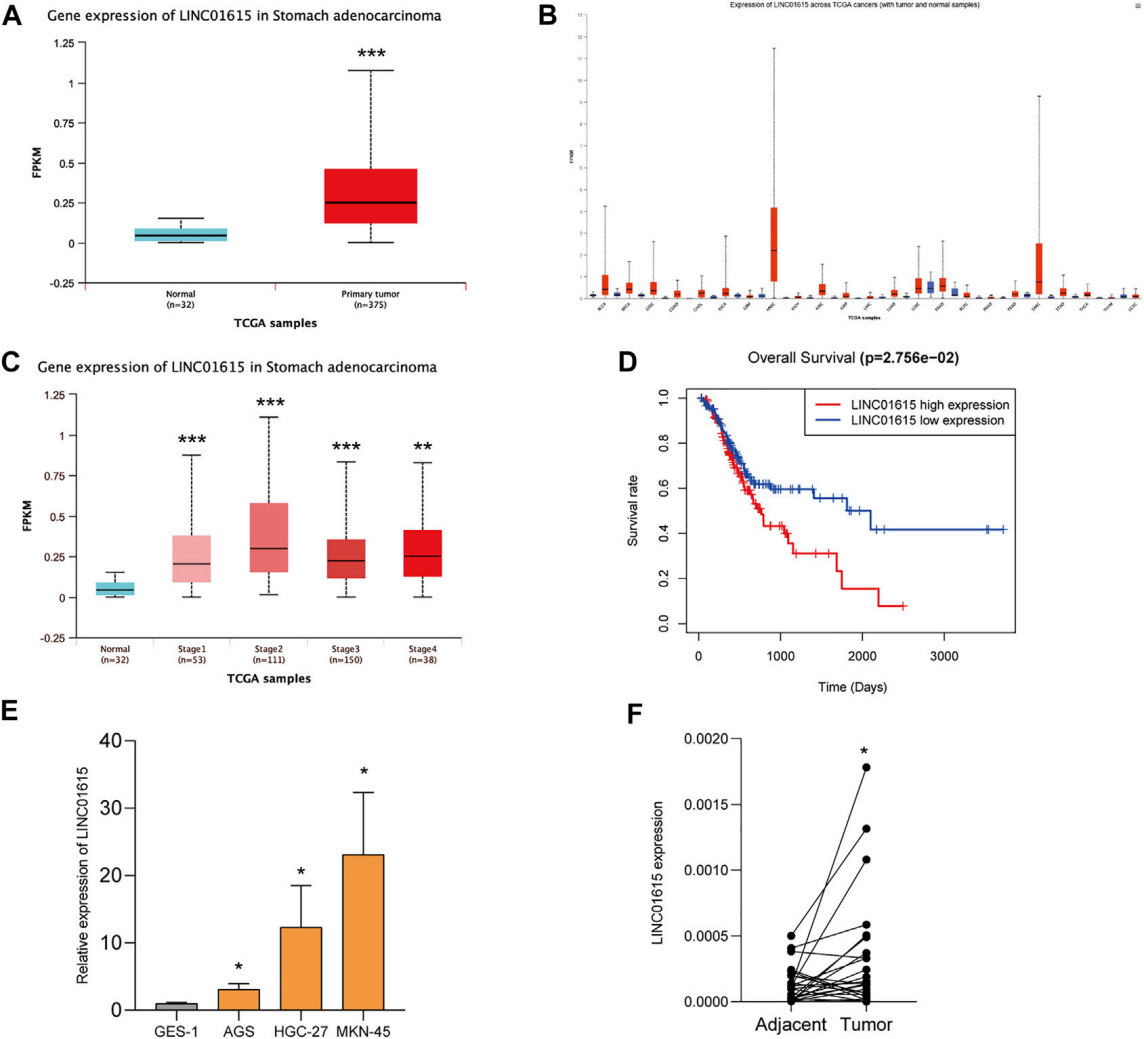
LINC01615 is upregulated in STAD. **(A)** UALCAN analysis revealed that LINC01615 is upregulated in STAD tumor tissues. **(B)** Expression of LINC01615 across TCGA tumors (with tumor and normal samples). **(C)** LINC01615 levels in different TNM stages (UALCAN). **(D)** High LINC01615 expression predicted poor overall survival in STAD patients in the entire dataset. **(E)** LINC01615 was upregulated in gastric cell lines. **(F)** LINC01615 was upregulated in gastric cancer tissues. *p < 0.05, **p < 0.01, ***p < 0.001.

The UALCAN database revealed that the expression of LINC01615 is significantly elevated in STAD tissues (Figure 6A). In addition, UALCAN program analysis also showed that the expression of LINC01615 in diverse cancer types such as bladder carcinoma (BLCA), head and neck squamous cell carcinoma (HNSC), and sarcoma (SARC) was notably upregulated (Figure 6B). The relative levels of LINC01615 across stages 1–4 of STAD were also analyzed using the UALCAN database (Figure 6C). Compared to normal condition, all four stages highly expressed LINC01615, showing a statistical significance (*p* < 0.05). High LINC01615 expression also correlates with the overall survival of STAD patients in the entire dataset (Figure 6D).

We then detected the expression of LINC01615 in the normal gastric mucosa epithelial GES-1 cell line and gastric cancer cell lines (AGS, HGC-27, and MKN-45) (Figure 6E). LINC01615 expression was significantly higher in gastric cancer cell lines than in the GES-1 cell line. Also, in 25 gastric cancer tissues and matched tissues adjacent to carcinoma, LINC01615 expression was higher in gastric cancer tissues (Figure 6F).

### LINC01615 Acted as ceRNA in Ferroroptosis

**FIGURE 7:**
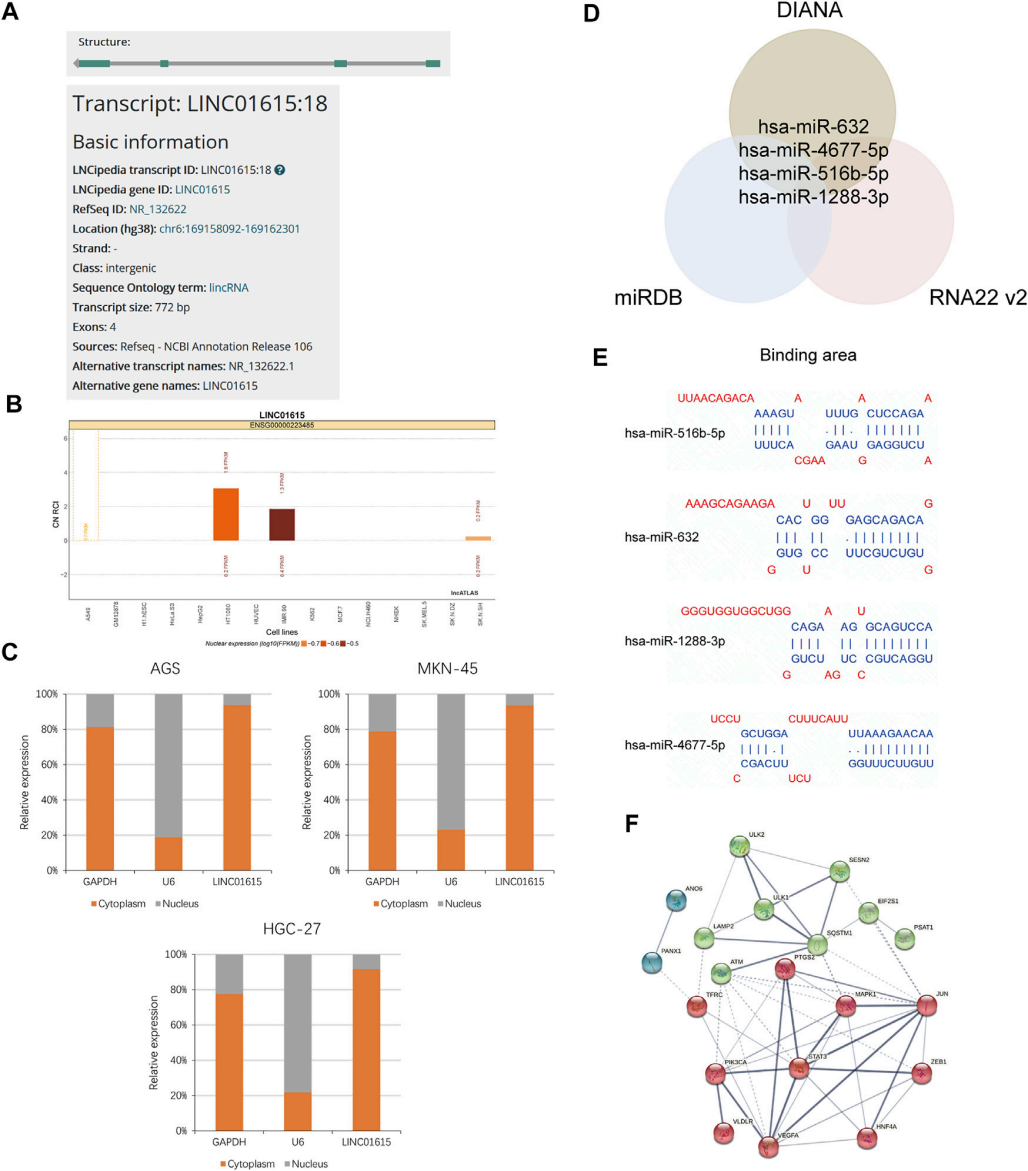
LINC01615 acted as a molecular sponge for miRNA in STAD. **(A)** Information about LINC01615 in LNCipedia. **(B)** Subcellular localization for LINC01615 in lncATLAS. **(C)** Subcellular fraction assay. **(D)** Potential miRNA targets for LINC01615. Intersection of the results from DIANA, miRDB, and RNA22 V2 prediction. **(E)** Depiction of the binding sequences of 4 miRNAs on LINC01615. **(F)** PPI network for 20 mRNAs related to ferroptosis with kmeans clustering.

Next, we analyzed how the LINC01615 contributes to the process of STAD in the molecular mechanism. First, we searched the information about LINC01615 in NCBI and LNCipedia and selected transcript variant 1 for further study (Figure 7A). Then, we analyzed the subcellular localization for LINC01615 in lncATLAS, which showed that LINC01615 is mainly cytoplasmic (Figure 7B). Also, we did a nuclear-cytoplasmic fractionation assay to confirm the result. We observed that in gastric cancer cell lines, most LINC01615 was enriched in the cytoplasm (Figure 7C). We then used bioinformatics databases (DIANA, miRDB, and RNA22 v2) to predict potential target microRNAs (miRNAs) of LINC01615. All the three databases contain four target miRNAs: miR-4677–5p, miR-516b–5p, miR-632, and miR-1288–3p (Figures 7D,E).

Subsequently, we figured out that the 26 mRNAs related to ferroptosis could be regulated by the four miRNAs mentioned above with miRDB, Targetscan, and DIANA-Tarbase. The 26 mRNAs related to ferroptosis are offered in Supplementary Table S4. Then, we obtained protein–protein interaction (PPI) networks for 26 mRNAs with STRING. We hid disconnected nodes in the network and divided the remaining 20 connected proteins into three clusters with kmeans clustering (Figure 7F). In addition, we explored the potential biologic function of LINC01615 with GSEA between the LINC01615 high-expression group and low-expression group of the TCGA-STAD project. Cell adhesion molecules, ECM receptor interaction, and the Hedgehog signaling pathway were enriched in the LINC01615 high-expression group (Supplementary Figure S7).

## Discussion

In this study, a three-ferroptosis-related-lncRNA (AP000695.2, AL365181.3, and LINC01615) model was constructed to predict prognosis for STAD patients. In the beginning, we filtered data and identified ferroptosis-related lncRNAs by co-expression networks. Then, three lncRNAs (AP000695.2, AL365181.3, and LINC01615) were selected to construct a predictive model according to the risk coefficients. Patients were categorized into a high- and a low-risk group by risk score. OS of STAD patients between high- and low-risk groups was statistically significant. The AUC result also indicated that the risk score has a good predictive ability. Cox analysis further confirmed that the risk score was an independent prognostic factor for STAD. Besides, this model was validated to have consistently good predictive ability in the validation dataset and entire dataset. Therefore, the three-ferroptosis-related-lncRNA model can predict the OS of STAD patients well.

Zha et al. have confirmed that AP000695.2 was highly expressed in STAD, which was associated with the tumor stage and distant metastasis (Zha et al., 2021). GO and KEGG analyses indicated that AP000695.2 was closely related to the development of tumor (Zha et al., 2021). AP000695.2 was not only an optimal diagnostic lncRNAs biomarker but also a prognostic lncRNAs biomarker for head and neck squamous cell carcinoma (Hu et al., 2020). Dong et al. proposed that LINC01615 affected the extracellular matrix and further impacted the metastasis of hepatocellular carcinoma (Ji et al., 2019). There are few reports on AL365181.3.

In this study, we found LINC01615 is upregulated in STAD tumor tissues and cell lines. A nuclear-cytoplasmic fractionation assay observed that in gastric cancer cell lines, most LINC01615 was enriched in the cytoplasm, which suggests that LINC01615 may exert its effects by ‘‘sponging’’ miRNA through competitive binding to miRNA, thereby preventing them from repressing their target mRNA. Then we predicted four potential target microRNAs (miR-4677–5p, miR-516b–5p, miR-632, and miR-1288–3p) in several databases and figured out the mRNAs related to ferroptosis that could be regulated by them.

MiR-1288–3p is reported to reduce the expression of CTNNBIP1, spurring cell migration in lung cancer (Xiao et al., 2021). It can also target RERG, the regulator of the Ras/ERK pathway, to control cell proliferation and apoptosis in colorectal cancer (Hou et al., 2020). Through negative modulation of GSK3β (Sun et al., 2020; Zhou et al., 2020), MYCT1 (Pu et al., 2019), Fascin actin-bundling protein 1 (FSCN1) (Ou et al., 2020), and TFF1(Shi et al., 2019), miR-632 modulated the WNT/β-catenin pathway and subsequently promoted cancer cell migration, invasion, proliferation, tube formation, and endothelial cell recruitment. MiR-516b–5p are reported to interact with ARHGAP5 (Yang et al., 2021), XBP1 (Huang et al., 2021), STAT3 (Xu et al., 2020), ITGA11 (Song et al., 2020), and AKAP2 (Lin et al., 2020).

In conclusion, we established a three-ferroptosis-related-lncRNA (AP000695.2, AL365181.3, and LINC01615) model that can predict the prognosis and provide a prognostic nomogram for the precision medicine of STAD patients. However, the interaction between ferroptosis and lncRNAs still has many unknown mechanisms that need to be explored. We expect to provide a promising target for LINC01615 for research in the future.

## Data Availability

The original contributions presented in the study are included in the article/Supplementary Material; further inquiries can be directed to the corresponding authors.
