# Supplementary material for: Establishment and Validation of a Ferroptosis-Related Long Non-Coding RNA Signature for Predicting the Prognosis of Stomach Adenocarcinoma
Source: Front Genet. 2022 Feb 15;13:818306. doi: 10.3389/fgene.2022.818306 (PMC8886230; doi:10.3389/fgene.2022.818306)
Supplement: Supplementary file 2 [file Table1.DOCX]

**Supplementary Figure**


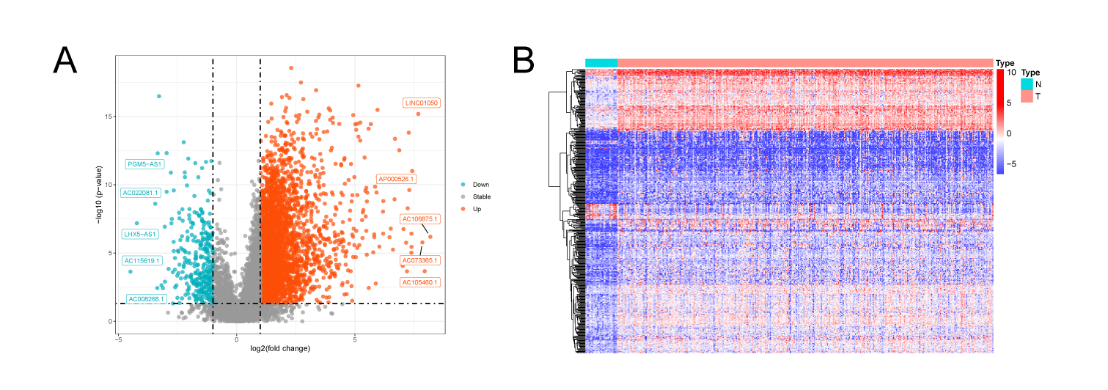


**Supplementary Figure 1:** Volcano plot and heatmap of ferroptosis-related lncRNAs of stomach adenocarcinoma samples in TCGA-STAD Project. (A) Volcano plot of lncRNAs in STAD samples from TCGA-STAD Project. (B) Heatmap of 414 ferroptosis-associated lncRNAs. N indicates non-tumor tissues; T indicates tumor tissues.

**
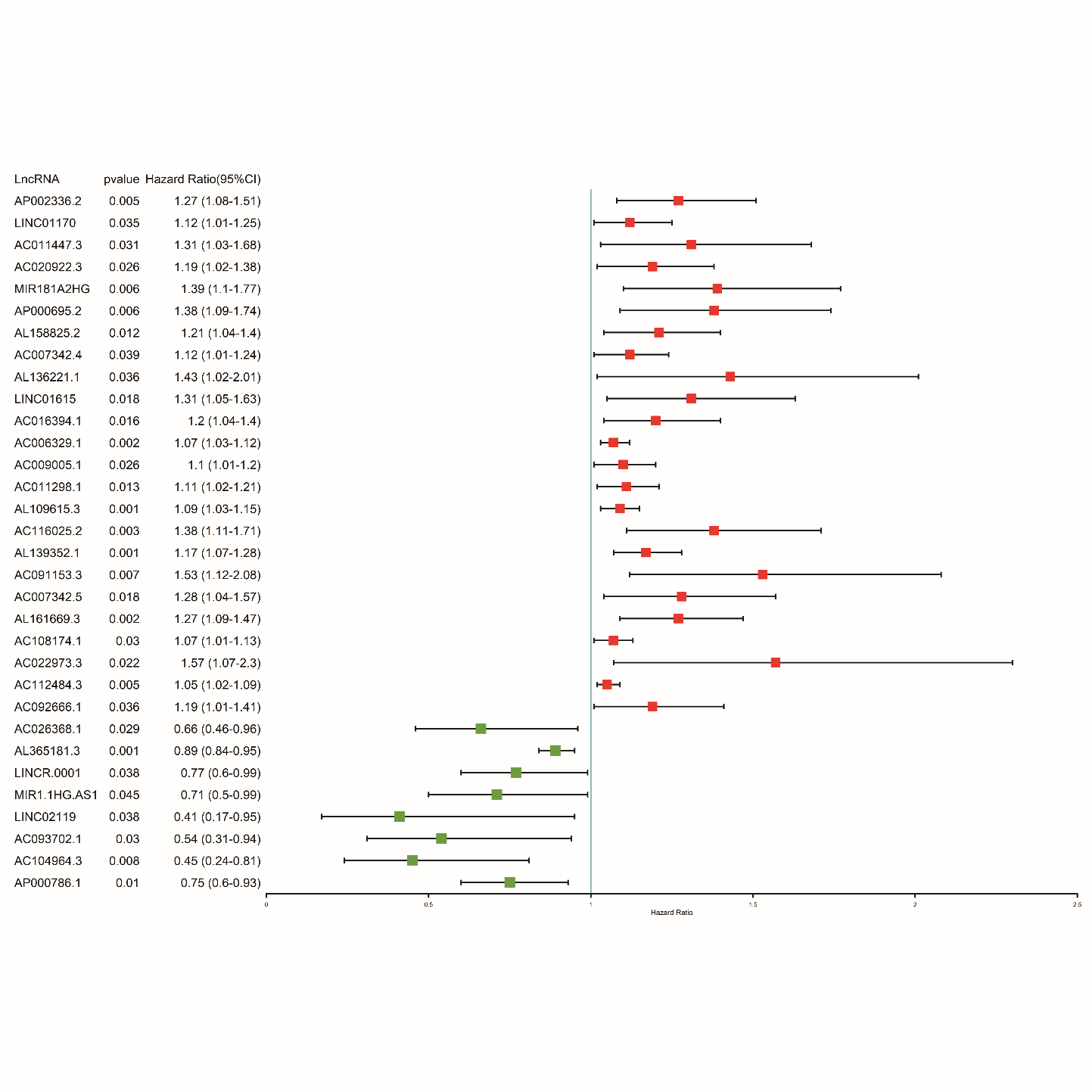
**

**Supplementary Figure 2:** Univariate Cox regression illustrated thirty-two ferroptosis-related lncRNAs associated with prognosis. Abbreviations: HR, hazard ratio; CI, confidence interval.


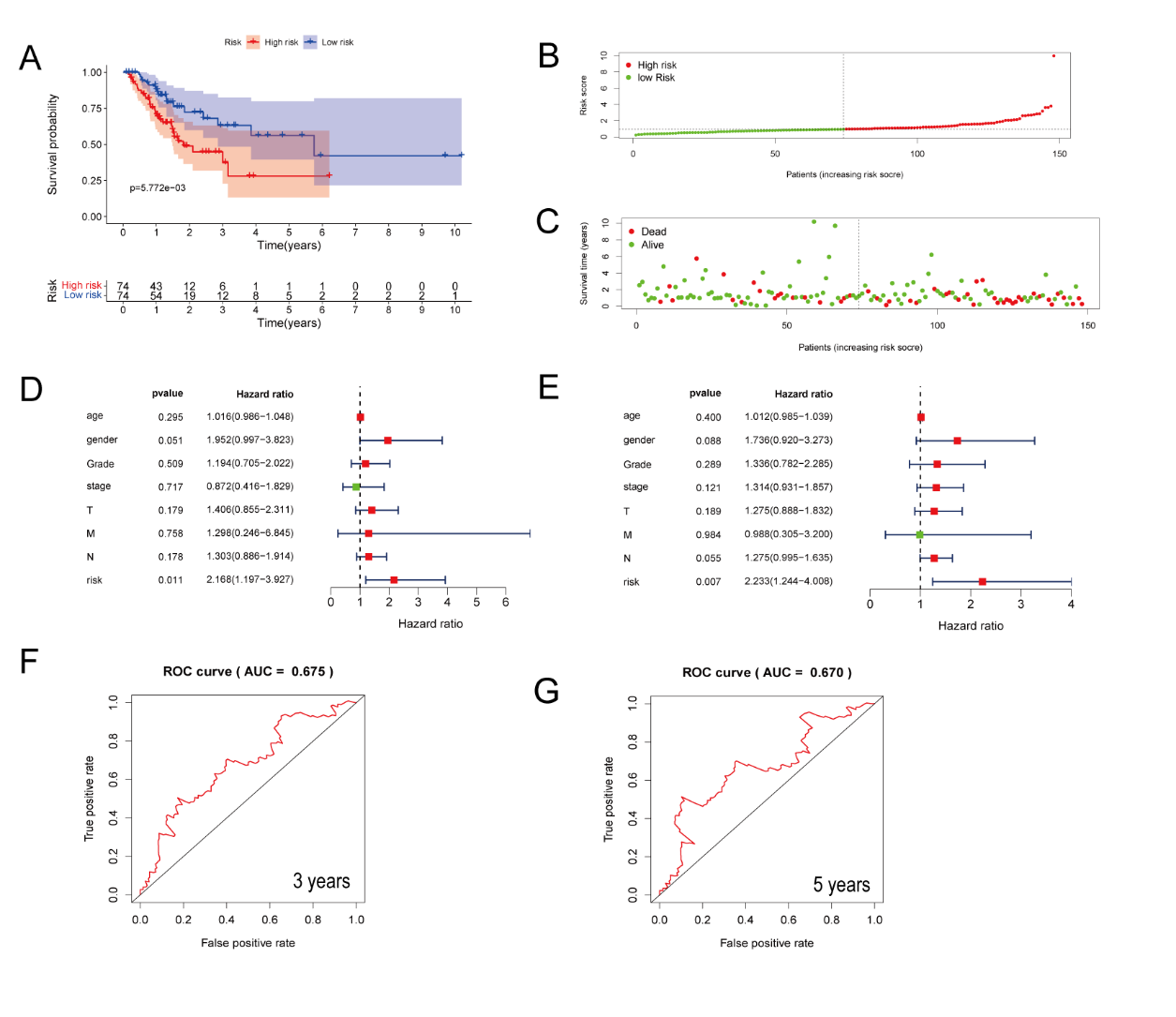


**Supplementary Figure 3: Identification and assessment of a three-ferroptosis-related-lncRNA model for predicting prognosis of STAD in the primary dataset. (A) Kaplan–Meier curves of OS in the high-risk and the low-risk groups in the primary dataset. (B) The distribution of patients in different risk groups. (C) Survival status of patients in the high-risk and the low-risk groups. (D) A forest plot of univariate Cox regression analysis in the primary dataset. (E) A forest plot of multivariate Cox regression analysis in the primary dataset. (F), (G) 3- and 5-year ROC curves illustrating the predictive accuracy of the model for prognosis in the primary dataset.**


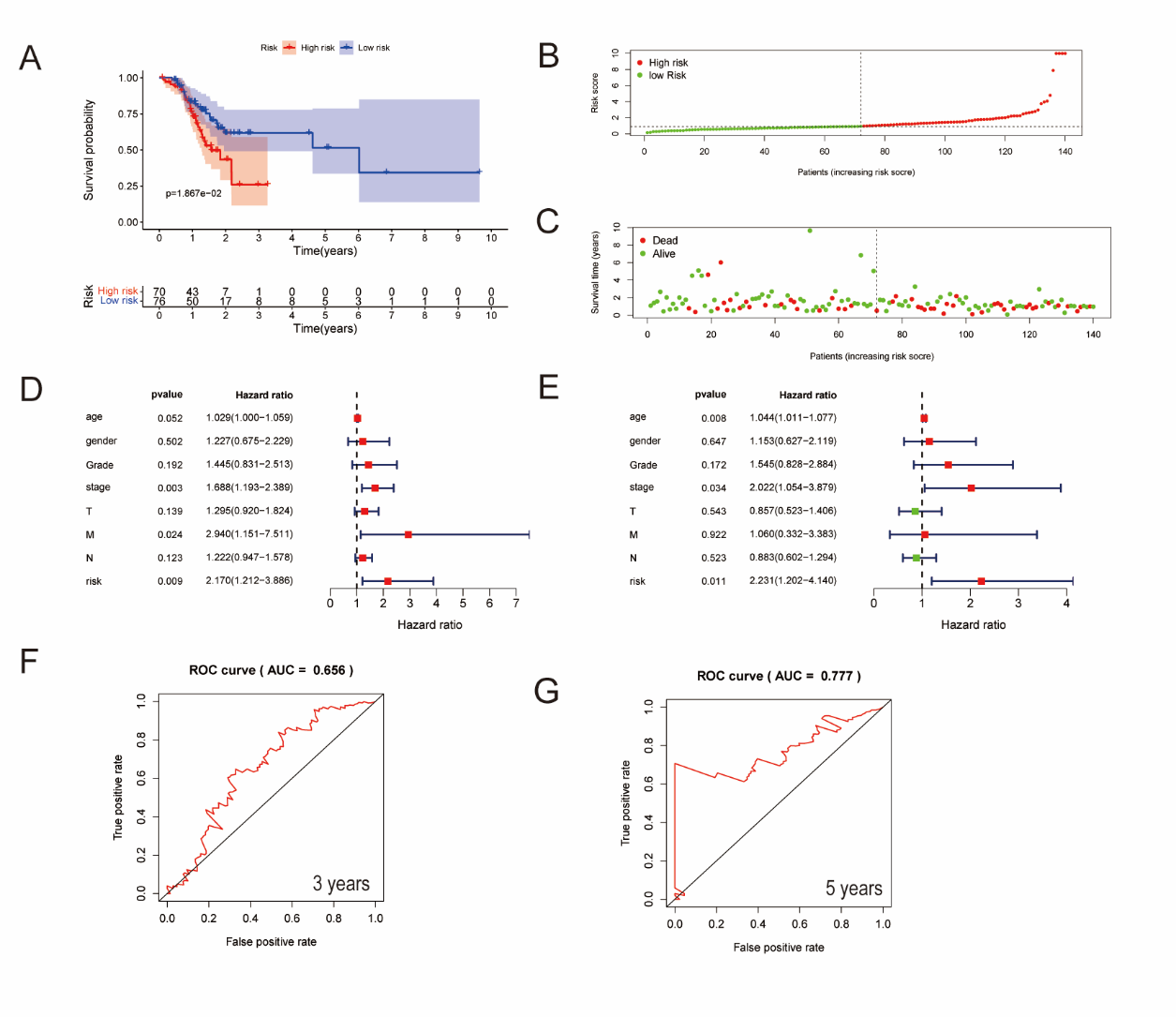


**Supplementary Figure 4: Validation of a three-ferroptosis-related-lncRNA model for predicting prognosis of STAD in the validation dataset. (A) Kaplan–Meier curves of OS in the high-risk and the low-risk groups in the validation dataset. (B) The distribution of patients in different risk groups. (C) Survival status of patients in the high-risk and the low-risk groups. (D) A forest plot of univariate Cox regression analysis in the validation dataset. (E) A forest plot of multivariate Cox regression analysis in the validation dataset. (F), (G) 3- and 5-year ROC curves illustrating the predictive accuracy of the model for prognosis in the validation dataset.**


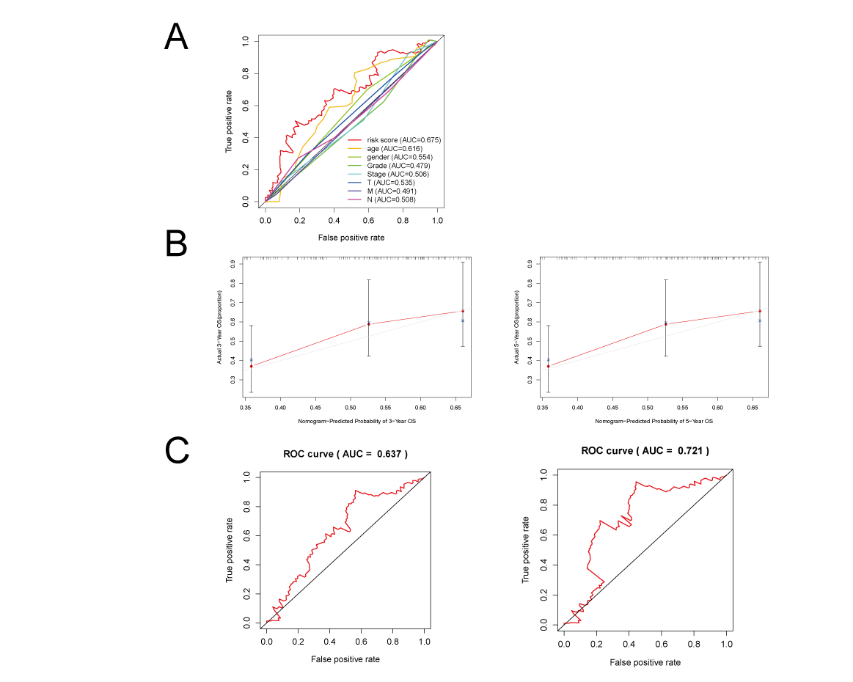


**Supplementary Figure 5: Evaluating predictive ability of the nomogram in the primary dataset. (A) ROC curve analysis of risk scores and other clinical risk factors in the primary dataset. (B) Calibration plots of the nomogram for predicting three- and five-year OS in the primary dataset. (C) Time-dependent ROC curves of the nomogram for predicting OS in the primary dataset.**


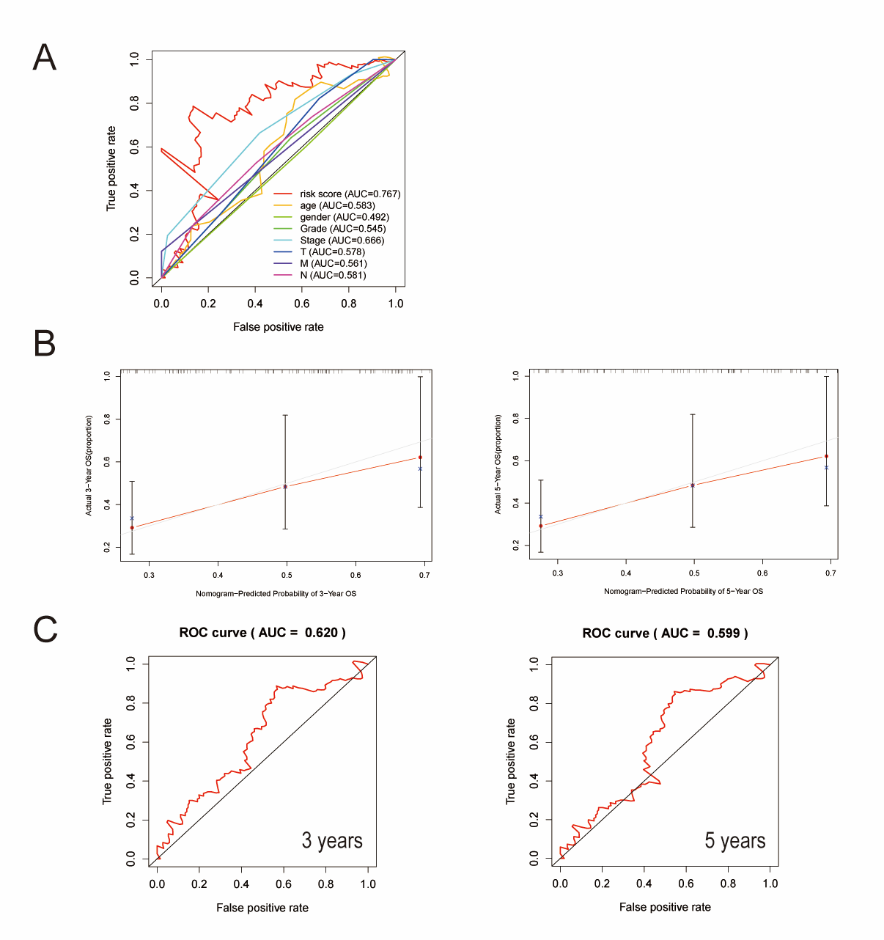


**Supplementary Figure 6: Evaluating predictive ability of the nomogram in the validation dataset. (A) ROC curve analysis of risk scores and other clinical risk factors in the validation dataset. (B) Calibration plots of the nomogram for predicting three- and five-year OS in the validation dataset. (C) Time-dependent ROC curves of the nomogram for predicting OS in the validation dataset.**


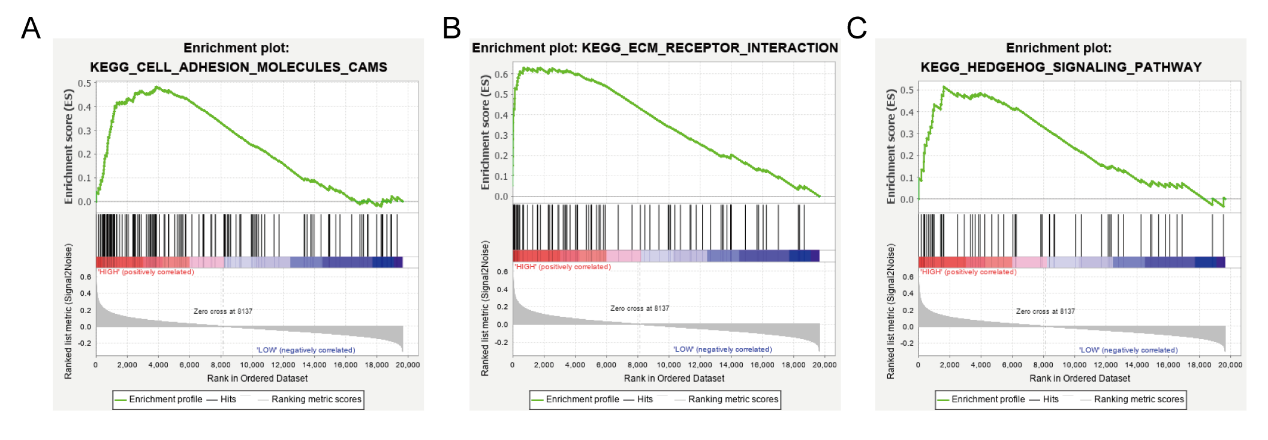


**Supplementary Figure 7: Gene set enrichment analysis between LINC01615 high expression group and low expression group. (A)** Cell adhesion molecules CAMs**, (B)** ECM receptor interaction**, (C)** Hedgehog signaling pathway **were enriched in the LINC01615 high expression group All |NES|>1, NOM p-val < 0.05, FDR q-val < 0.25.**


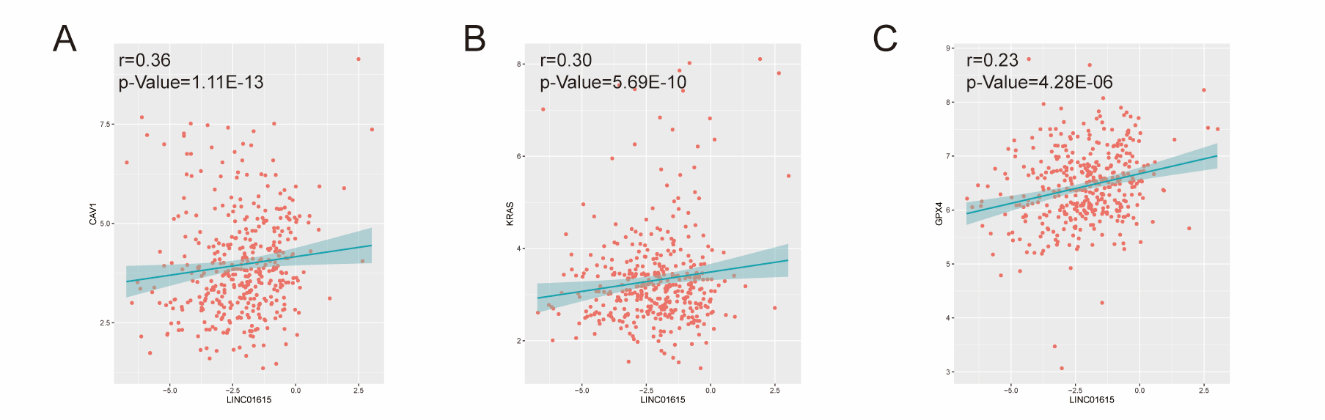


**Supplementary Figure 8: Co-expression of the LINC01615 and Ferroptosis-related protein in STAD. (A) CAV1, (B) KRAS, (C) GPX4.**
